# Design and implementation of a web-based, respondent-driven sampling solution

**DOI:** 10.1186/s12911-023-02217-0

**Published:** 2023-07-05

**Authors:** Catherine R. McGowan, Promise Ekoriko, Mervat Alhaffar, Sarah Cassidy-Seyoum, Steven Whitbread, Phil Rogers, Lucy Bell, Francesco Checchi

**Affiliations:** 1 Department of Infectious Disease Epidemiology and International Health, Faculty of Epidemiology and Population Health, London School of Hygiene and Tropical Medicine, Keppel Street, London, WC1E 7HT UK; 2 Information Technology Services, London School of Hygiene and Tropical Medicine, Keppel Street, London, WC1E 7HT UK

**Keywords:** Respondent-driven sampling, Mortality estimation, webRDS, Hard-to-reach populations, Chain-referral, Yemen

## Abstract

**Background:**

Respondent-driven sampling (RDS) refers both to a chain-referral sampling method and an analytical model for analysing sampled data. Web-based respondent-driven sampling (webRDS) uses internet-based recruitment coupled with an electronic survey to carry out RDS studies; there is currently no commercially available webRDS solution. We designed and developed a webRDS solution to support a research study aimed at estimating conflict-attributable mortality in Yemen. Our webRDS solution is composed of an existing survey platform (i.e. ODK) and a bespoke RDS system. The RDS system is designed to administer and manage an RDS survey cascade and includes: (1) an application programming interface, (2) a study participant client, and (3) an administrator interface. We report here on the design of the webRDS solution and its implementation.

**Results:**

We consulted members of the Yemeni diaspora throughout the development of the solution. Technical obstacles were largely the result of: WhatsApp’s policies on bulk messaging and automated messaging behaviour, the inherent constraints of SMS messaging, and SMS filtering behaviour. Language support was straight-forward yet time consuming. Survey uptake was lower than expected. Factors which may have impacted uptake include: our use of consumable survey links, low interest amongst the diaspora population, lack of material incentives, and the length and subject matter of the survey itself. The SMS/WhatsApp messaging integration was relatively complex and limited the information we could send potential participants.

**Conclusion:**

Despite lower-than expected survey uptake we believe our webRDS solution provides efficient and flexible means to survey a globally diverse population.

**Supplementary Information:**

The online version contains supplementary material available at 10.1186/s12911-023-02217-0.

## Background

Respondent-driven sampling (RDS) is a chain-referral sampling approach that was developed as a means of obtaining survey data from hard-to-reach populations for which conventional sampling frames are difficult to establish [1]. The chain-referral begins with a convenience sample of ‘seed’ participants who are recruited from within the target population. Seed participants are invited to complete a survey and then invite others who meet the recruitment criteria from within their social networks. All participants are encouraged to issue onward invitations to a pre-defined number of participants. The survey cascades until the target enrolment has been reached. Each participant is identified though a unique identification number (UID) as well as the UID of the person from whom they received the invitation. An RDS survey also requires that each participant provide an estimation of their personal network size which is used to define the overall network structure and reduce selection bias in study population estimates. Despite the lack of a defined sampling frame and the initial purposive seed recruitment, various applications of RDS have been shown to yield samples that can be adjusted to account for non-random recruitment to produce robust population estimates [2, 3]. Unlike other chain-referral methods (e.g. snowball sampling) the RDS survey population self-recruits without interference from the researchers. Peer-referral increases recruitment of research participants who are unlikely to come into contact with researchers due, for example, to a desire not to be identified as part of a peer-group, or a reluctance to engage with researchers or others outside of their peer group. Peer-referral methods can also be designed to offer participants complete anonymity, even from the research team. The momentum of an RDS survey is sometimes maintained by providing material incentives, both for completing the survey, and for each downstream recruitment [4].

Early applications of RDS required face-to-face interactions both to cascade the survey and to collect survey responses. Increasingly, RDS is conducted online to simplify recruitment, expand geographic coverage, and improve survey uptake [5]. Internet-based RDS (a.k.a. webRDS) has been used to survey typically hard-to-reach populations including: migrant populations [6], men who have sex with men [7, 8], people who use drugs and alcohol [9, 10], and people with precarious employment [11]. There is currently no commercially available solution for managing webRDS though several *ad hoc* approaches have been described in the literature [7–9, 11–15]. These webRDS solutions vary considerably in terms of the degree of automation within the RDS process, as well as the extent to which the system was able to provide anonymity to participants. However, none of the available sources provide a thorough description of the RDS system architecture making it difficult to determine the strengths and weaknesses of each design.

A 2021 scoping review of the applications and recruitment performance of webRDS suggested that webRDS could be enhanced by offering multiple recruitment options (e.g. WhatsApp, SMS, email), although it was not possible to empirically demonstrate how these enhancements impacted recruitment performance [5]. Notably, the review highlighted a key paradox of webRDS: though RDS is meant to improve access to hard-to-reach populations, webRDS is not a suitable solution for those populations that are hard-to-reach due to high levels of digital exclusion [5].

### Estimating conflict-related mortality in Yemen

As a result of ongoing conflict, Yemen is experiencing a protracted crisis with nearly three quarters of the population currently in need of humanitarian assistance [16]. Most Yemenis are exposed to active conflict, food insecurity, and/or lack of access to essential services. The absence of data on conflict-related mortality makes it difficult to understand the impacts of the current crisis. Logistical constraints and security risks render field studies infeasible, and the Yemeni vital events registration service is understood to have limited functionality. During scoping work with Yemeni stakeholders, we identified active networks within the Yemeni diaspora in different countries and hypothesised that these networks could be surveyed through RDS to collect information on mortality amongst family members residing in Yemen. We elected to use an RDS methodology as it would allow us to base population estimates on a theoretically robust, probabilistic sample that could be weighted to account for non-random recruitment. Given the geographic diversity of the Yemeni diaspora, the sensitivity of the research topic (requiring strict participant anonymity), and the sample size requirements (we estimated that a minimum sample of 1200 participants would be needed to detect a 30% increase in mortality from baseline), we elected to use a webRDS approach for this study. The findings from the diaspora survey are published elsewhere [17].

As we were unable to identify an existing webRDS platform we opted to develop a bespoke webRDS solution that was: appropriate to our study aims and objectives, sensitive to the ethical obligations of carrying out research in this population, informed by the findings of the aforementioned 2021 scoping review [5], and guided by principles of digital development [18]. Though our focus was on developing the webRDS solution to support the Yemen mortality study, we designed the solution with the intention of making it available as a system-agnostic, open-source, global good. The objective of this paper is to describe the development, deployment, and informal evaluation of our webRDS solution.

## Implementation

### Study requirements

Our sampling frame included any member of the Yemeni diaspora aged 18–49 years. We assumed a moderate to high degree of digital literacy within our study population. We also assumed that potential participants would be more likely to complete the survey if they were able to do so on their platform of choice (e.g. mobile device or computer). Informed by Helms et al., we assumed that participants would be more inclined to engage in onward recruitment if they were able to do so using the preferred means of communication (e.g. email, WhatsApp, SMS) of eligible participants within their networks [5]. To increase accessibility we aimed to make all user-facing content available in both English and Arabic. As the RDS methods requires reconstruction of the survey cascade, we needed to create consumable invitation links and generate associated metadata indicating the identity (based on an anonymous, auto-generated unique identifier) of the participant, and the identity of the participant who invited them. Finally, we aimed to ensure that the solution was intuitive and that it demonstrated adherence to common accessibility standards (i.e. the Web Content Accessibility Guidelines 2.1) [19].

#### Ethics and governance

Given the political nature of the ongoing conflict in Yemen we needed to allow participants to remain anonymous (even to the study team). As the survey was part of a research project, and because it had the potential to cause emotional distress, participants were required to provide informed consent through clear affirmative action. The survey included sensitive questions about deaths within the participant’s immediate family requiring careful thought about how, and at what point in the process, participants would be informed about what participation in the study would involve. A 2017 study by McGowan et al. concluded that most study participants find online consent, embedded in a web survey, to be an acceptable option for uncomplicated and low risk studies [20]; thus, we elected to incorporate the consent process into the survey itself.

#### System requirements

Due to time and budget constraints we did not consider developing an entirely bespoke solution, opting instead to identify an existing survey platform, and to develop an RDS system to administer the survey and manage the survey cascade. We required a survey platform that was: customisable (and written in a common programming language), able to administer surveys in multiple languages, locally hostable, and secure. In addition, the survey platform needed to have a public application programming interface (API) with a thorough API specification. The RDS system required: user authentication, customisation of user-facing content (including multi-language support), user defined survey endpoints and/or the option to manually stop the survey, basic visualisations to monitor survey progress, and automated reminders.

### System description

**Fig. 1:**
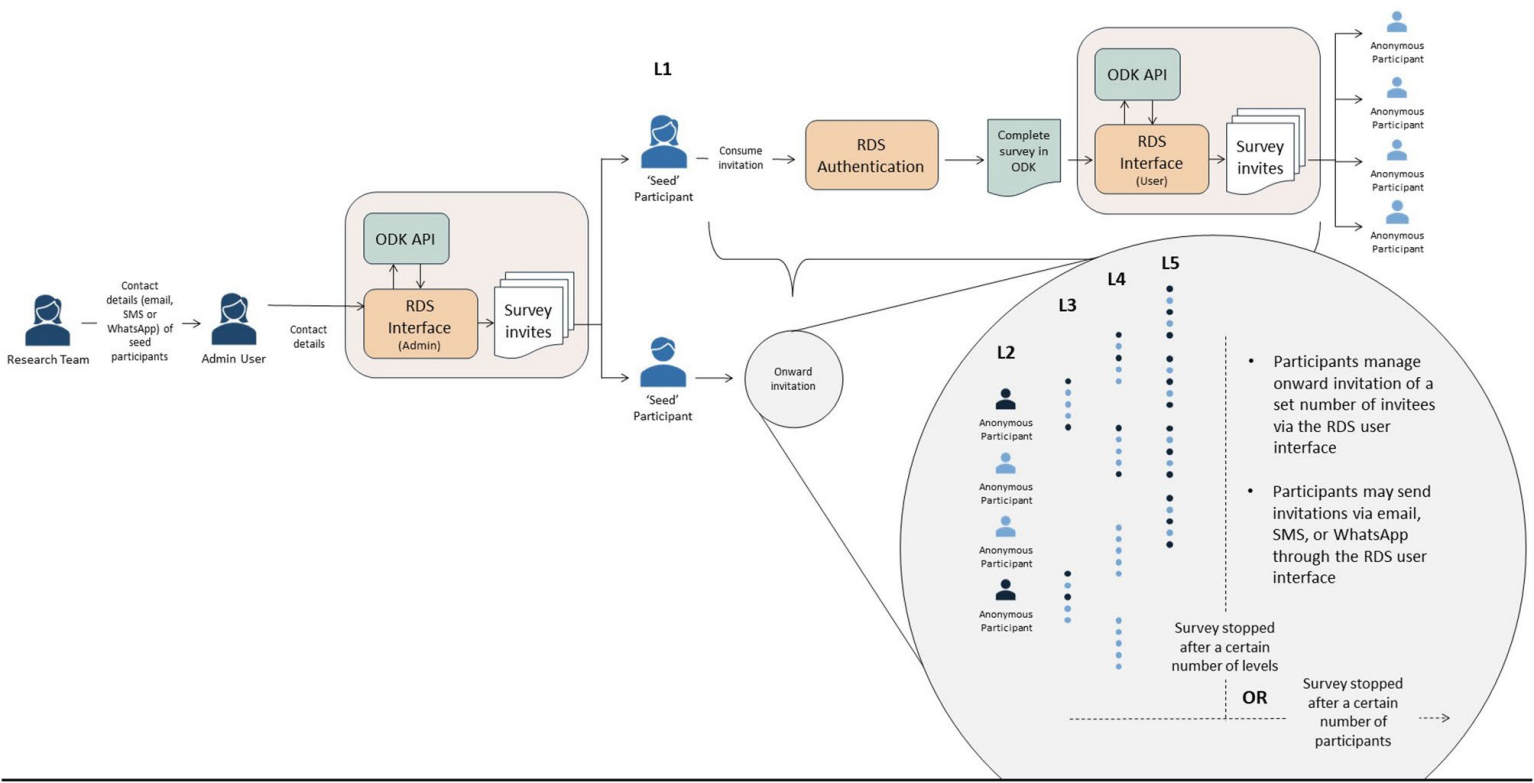
The RDS solution

The *RDS solution* included two distinct elements: (1) a survey platform, and (2) an *RDS system* (to administer the survey and manage the survey cascade). The RDS solution user journey is presented in Fig. 1.

#### The survey platform

As we set out to develop a solution based on Principles for Digital Development, we were committed to leveraging existing applications where possible [18]. ODK (https://getodk.org/) is a mature, extensible, open-source, platform-independent software for developing, deploying, and managing mobile data collection. ODK was an appealing option as: it is based on a common markup language (i.e. XML), it has a system API (allowing us to control most system behaviours) with a well-documented API specification, and it has an engaged and knowledgeable community of users. In addition, ODK has been institutionalised in our university. From a user perspective ODK was appealing largely due to its reliance on Enketo lightweight web-forms. Enketo is stable in a cross-hardware/cross-platform environment and has an accessible and intuitive user interface. Both ODK and Enketo support multi-language forms which was an important consideration as our survey needed to be available in both English and Arabic. We installed a siloed instance of ODK Central (Version 1.3.3) to allow us to manage ODK updates separate from the shared instance. The ODK API was used to generate a single-use, consumable link to access the ODK survey, and to provide information to the RDS system about survey status (i.e. started, completed).

#### The RDS system

We developed a multi-application, web-based RDS system consisting of a bespoke RDS API (to automate the survey cascade), an administrator interface (including multi-language survey customisation, manual control of the survey cascade, and basic visualisations), and a participant client (allowing participants to manage onward invitations).

**Fig. 2:**
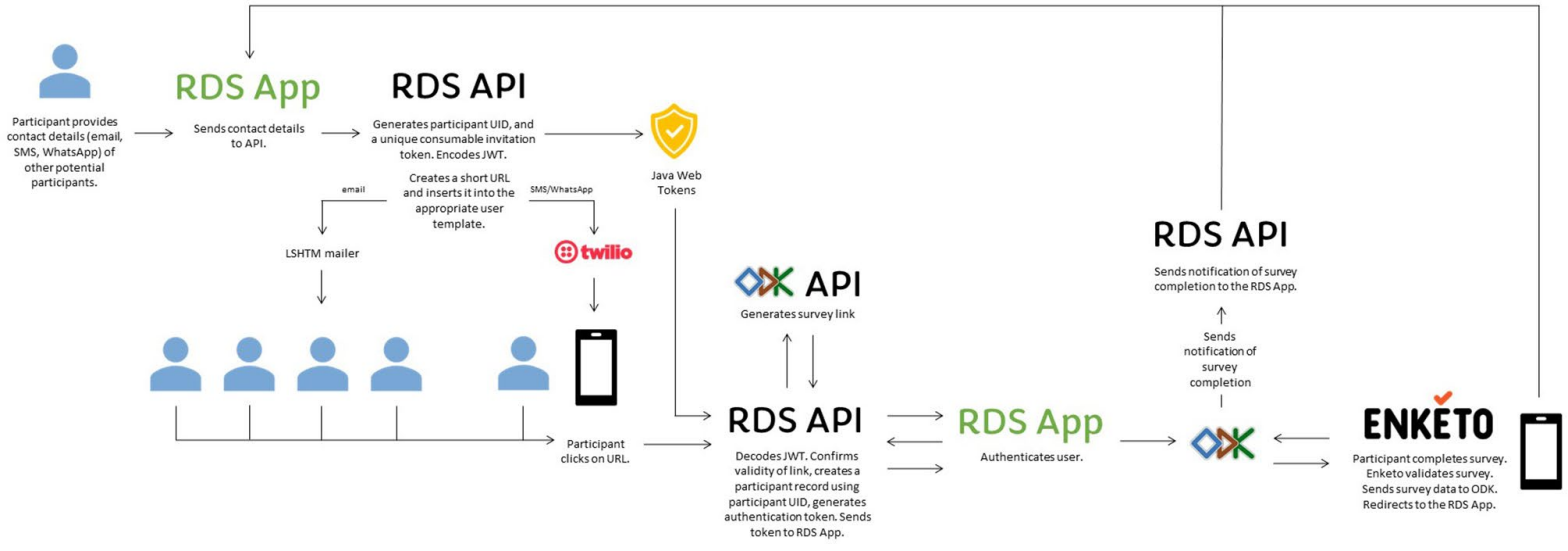
Invitation behaviour

The RDS system was built to manage the survey cascade by creating single-use invitation links (encoded using JSON Web Tokens) and through URL redirection to shift between the RDS solution and ODK (see Fig. 2). Participants who completed the survey were redirected by Enketo to the RDS participant client which allowed them to enter contact details for a predetermined number of potential participants for onward invitation. When the survey cascade is stopped - either based on predetermined parameters, or manually by the system administrator - the RDS system redirects to a terminal webpage.

The RDS API was designed to interface with the ODK API and other third-party applications including Twilio (https://www.twilio.com/) and Let’s Encrypt (https://letsencrypt.org/), and to relay emails to our internal mail system/SMTP servers. We used the Twilio web service API for managing outgoing SMS text messaging and for interfacing with the WhatsApp Business API for outgoing WhatsApp messaging. We used Let’s Encrypt for providing encrypted transport layer security (TLS) and secure socket layer (SSL) certificates for the RDS solution and for the ODK instance.

The RDS solution was developed using PHP programming language and was built with the Laravel framework. The solution supports PHP 7.4 and above. Authentication for system administrators was managed by the LSHTM lightweight directory access protocol (LDAP) directory services. All non-commercial packages were downloaded from Composer (https://github.com/composer/composer); permissions for Composer are unrestricted without limitations on the right to use, copy, modify, merge, publish, distribute, sublicence, and/or sell [21]. All third-party packages and their functions are listed in the Supplemental Materials.

#### Hosting

The RDS API, ODK Central, and other third-party applications are hosted on a virtual LSHTM SUSE server (version 15.3), running Apache Web Server (version 2.4), Docker (version 20.10.9-ce), and Docker Compose (version 1.29.2).

### Information security

The RDS system uses encryption to secure data transfer between the various solution components. We used TLS and SSL protocol encryption between the server and the client browser using LetsEncrypt. All internal communication between components and third-party providers was carried out via SSL. The RDS system uses local key-based encryption to encode participant contact information (email addresses and phone numbers) – contact information is secured both during transit and at rest. The system was able to prevent duplicate invitations (by system administrators) by hashing (and comparing) the encrypted contact information.

The mortality survey was designed not to elicit or allow (e.g. through free text fields) participants to enter any potentially identifying personal data. The RDS system was designed to offer non-seed participants near-complete anonymity to the study team. Though participants were required to enter a phone number or an email address to invite others, there is no way for the system administrator to access contact details via the RDS dashboard. The developer and one member of the study team were only able to access phone numbers via Twilio’s user console (through messaging and error logs). Only the developer could access email addresses (by writing a script to decrypt the database values). The system was designed not to allow invitation of the same seed participant more than once and would notify the system administrator if a participant had previously been invited. However, in order to protect the identify of participants, the participant client was designed not to notify participants if they entered the contact details of someone who had already been invited. Whilst this created a risk of duplicate entries, we felt the likelihood of someone completing the survey twice, particularly in the absence of material incentives, was very low.

## Results

### Designing with the user

We consulted contacts in the Yemeni diaspora during the development of the RDS solution. In response to feedback we designed the RDS participant client and the ODK survey to default to Arabic (with the option for the participant to switch to English). We also sent all invitations (email, SMS, WhatsApp) in both English and in Arabic. In addition to including a link to the survey, email and WhatsApp invitations included a link to a study information webpage [22].

### Technical obstacles

#### WhatsApp messages via Twilio

Twilio does not automatically enable Twilio numbers for sending WhatsApp messages. Twilio requires the completion of an online application (which requires a Facebook (now Meta) Business Manager account) before it will grant access to the WhatsApp Business API. WhatsApp does not automatically allow bulk messaging or automated messaging behaviour; thus, all outgoing messages require pre-approval by WhatsApp as per their WhatsApp Business Messaging Policy [23]. This approval process – which was taking several days per message – complicated the iterative development of our communications. However, WhatsApp templates allow clickable ‘call to action’ buttons that appear as clickable buttons below the message which we used to link to a webpage containing study information. Though WhatsApp is not available in all countries it is a common application in the countries in which much of the Yemeni diaspora reside.

#### SMS

The character limit constraints for SMS messaging limited the amount of information we were able to provide in each message, particularly as Arabic script requires Unicode encoding and is therefore limited to 70 characters. Though some countries and/or user devices support concatenated SMS, lengthy unconcatenated survey invitations, in both languages were felt to be potentially confusing; thus, we opted for shorter SMS messages. It was not possible to include a link to the study website in SMS messages (as we did with WhatsApp messages); however, the link is included in the RDS participant client. Finally, both the US and Canada use SMS filtering to disallow application-to-person messaging from 10-digit phone numbers. As our Twilio account was registered in the UK, the phone number we were assigned was 10-digits. To sidestep this problem we requested a US number and redirected to it to successfully message potential participants on US and Canada-based telecommunication carriers. We were also required to register for the A2P 10DLC programme to ensure compliance within the SMS messaging ecosystem.

#### Language support

To avoid delays in deploying the survey we were unable to create a customisable participant client which would allow the system administrator to add language translations (and to modify the default English text). Instead, we built the Arabic translation into the participant client with the intention of creating an agnostic solution for future surveys. Managing right-to-left (RTL) text on participant facing webpages was time-consuming, but uncomplicated. Multi-language support in ODK is native to the ODK solution; however, our use of nested repeat groups in the survey itself required additional programming to accommodate gendered pronouns and adjectives in Arabic. We used PO Editor (https://poeditor.com/) as a software localisation management platform to manage translation of HTML documents.

### Survey uptake

We deployed the first RDS invitations to seed participants in early March 2022 and closed the survey in August 2022. The survey was completed by 93 participants and reached the fourth level (seed participants were level one, their invitees were level two, etc.). Most participants chose to send the survey to others using WhatsApp (n = 77, 59% of participant-issued invitations). Of the 166 individuals who received an email invitation just under half accepted (i.e. clicked on the invitation link), and 41% of those who had been invited completed the survey (Table 1). Notably, all participants were given five ‘tokens’ – meaning they could provide the RDS system with the contact details of up to five potential participants – yet none of the participants who used all five tokens achieved full downward recruitment.

**Table 1:** Survey uptake

	Invitations	Acceptance	Completion
Email	166	80 (48%)	68 (41%)
SMS	7	1 (14%)	0
WhatsApp	111	36 (32%)	25 (23%)

Of the 111 individuals who received an invitation via WhatsApp just over a third accepted, and 23% completed the survey. Only seven invitations were sent via SMS, only one accepted, and none completed the survey.

## Discussion

### Low uptake and limited survey cascading

Though we have yet to formally evaluate our solution we received informal participant feedback (via seed participants) suggesting that the RDS solution was intuitive and easy to use, with the exception of our use of consumable web links. We included a warning that the link was only clickable once in the invitation email but were unable to include a warning in the SMS messages (due to character limitations), or in the WhatsApp messages (out of a desire to limit the length of messages). Feedback indicated that some participants had clicked on the link, closed their browser, and then could not re-open the survey. As the system was designed to prevent the system administrator from sending repeat invitations using the same contact details (to prevent duplicate survey responses), we could not re-send invitations at the request of seed participants without changing the invitation medium. Given that across all invitation modes a total of 24 participants clicked on the invitation but did not complete the survey, we may need to consider modifying the RDS solution to ‘consume’ links only once the survey is complete.

We did not offer material incentives for completing the survey primarily due to budget limitations and based on our assumption that members of the diaspora would be eager to complete a survey with the potential to draw attention to the consequences of conflict in Yemen. Though the RDS solution could be adapted to issue material incentives in the form of gift cards, most gift cards can only be redeemed by the country-specific platform from which they are issued; thus, material incentives are likely to be better suited for geographically contained populations. However, we were concerned that a material incentive could motivate non-eligible participants to complete the survey or incentivise participants to complete the survey more than once. Duplicate and/or dummy survey data would complicate an accurate reconstruction of the survey cascade.

We also considered the possibility that the low uptake may have been a feature of our study population - rather than a limitation of the method. Our assumption that the diaspora shares a motivation to draw attention to the humanitarian need in Yemen may not have been correct (or fair). Historically Yemen has experienced multiple conflicts resulting in distinct diaspora cohorts. It may be the case that some potential participants were removed from, and less invested in, the current crisis and were therefore not motivated to complete the survey. It is also plausible that, despite providing clear assurances of the survey’s anonymity, some potential participants may have mistrusted the aims of the survey, or the extent to which contact information would be managed securely. Whilst Helms et al. 2021 note that the evidence points towards the use of material incentives to increase participation, the authors acknowledge that the data are limited and that the success of peer recruitment methods for delivering interventions often relies on participants’ affinity towards the intervention and its anticipated or experienced outcomes [5]. Notably, studies of diaspora engagement in health system strengthening found that individuals in the diaspora are considerably less likely to engage in activities in support of their country of origin if they have no intention of returning [24].

Finally, we received participant feedback (via seed participants) that the survey was lengthy for those with large families. Some participants also reported finding the survey topic distressing and may therefore have been disinclined to complete it. One participant indicated her reluctance to forward the survey to friends who she knew to have suffered traumatic bereavement. In response to this feedback we have added a metadata field in the RDS solution which allows the system administrator to provide a link to a webpage including mental health resources (as well as some default framing text). The use of an RDS solution to deliver and manage a web survey, *and* to disseminate mental health resources may help to mitigate against any potential distress.

### SMS and WhatsApp

The complexity of developing and supporting SMS/WhatsApp messaging is difficult to justify given the relatively small proportion of participants who completed surveys when invited in this manner; however, preferences around modes of communication are very context specific and the availability of SMS/WhatsApp may be critical in other settings. In retrospect, our scoping work ought to have determined the communication preferences of our diaspora population. Ultimately, Enketo is designed to deliver web forms via mobile device; thus, it might have been more straightforward to limit invitations to email whilst noting in the email invitation that participants may launch the survey on a mobile device. However, we suspect that issuing onward invitations may have been more complicated on a mobile device as participants would either need to know the contact details of those to whom they wished to send the survey, or they would need to switch applications (up to five times) to locate the required contact details. In the end this may not have been a useful feature; however, we intend to carry out an evaluation of the solution to evidence this assumption. Finally, the character limits of SMS messaging, and the desire to limit the amount of information in WhatsApp invitations, meant that we could not provide participants with as much information in mobile invitations as we could in an email which may have reduced potential participants’ willingness to participate. However, one unexpected advantage of supporting SMS/WhatsApp using Twilio was that we were easily able to monitor messaging failures.

### Sustainability

We designed the RDS solution to be sustainable by ensuring the solution was lightweight, written in a common programming language(s), and versatile (e.g. the RDS solution can be used with multiple survey platforms and/or any directory management system). In addition, the solution leverages the sustainability of ODK Central, Twilio, SMS, and WhatsApp. However, integration with third party platforms can render a solution vulnerable to disruption when third party applications are updated. For example, our update from ODK Central 1.3.3 to 1.4 – which was necessary to address issues related to the conversion of our XLSForm to ODK XForms – caused a persistent certificate error likely caused by our server configuration. We have deposited the source code for the API (https://bitbucket.org/lshtm-public/rds-api/src/master/) and the user interface (https://bitbucket.org/lshtm-public/rds-ui/src/master/) in Bitbucket (https://bitbucket.org) under a GNU General Public License to allow further development and continuous improvement by other researchers.

### Limitations

We have not yet carried out a formal evaluation of our RDS solution. Whilst we received feedback via our seed participants - we could not follow up with our participants directly as their participation was anonymous - we are likely missing feedback from key groups (e.g. those who did not accept the survey invitation, or those outside our eligibility criteria). As the survey was anonymous, we are unable to identify pattens in survey uptake. Additional mortality surveys that we are currently planning may provide us with the opportunity to evaluate the RDS solution in addition to determining the feasibility of conducting mortality surveys in this manner.

## Conclusion

In the absence of a commercial webRDS platform we were able to develop a fit-for-purpose solution to manage RDS survey invitations. Our solution included multi-language support, integration with ODK Central, and allowed survey invitations to be sent through email and SMS/WhatsApp services. Though the survey did not cascade to the extent that we had hoped, we received 93 completed surveys which include data describing the status of 1, 704 unique individuals. Informal feedback suggests that though completing the mortality survey was time consuming for some participants, and was upsetting to others, the RDS solution was deemed accessible, and the survey was clear and easy-to-follow. Whilst the RDS solution can be easily adapted to carry out mortality surveys, it is broadly applicable to other types of surveys (e.g. health exposures and outcomes, knowledge and attitudes, service utilisation). We believe that had it been easier to galvanise our study population we may have improved uptake; nonetheless, we maintain that webRDS is a suitable approach from which to estimate mortality in complex settings.

## Electronic supplementary material

Below is the link to the electronic supplementary material.

Supplementary Material 1

## Data Availability

Project name: RDS Solution. Operating system(s): Platform independent. Node JS. Programming language: PHP. Other requirements: Node ^14/PHP ^7.3. License: GNU Lesser General Public License. Any restrictions to use by non-academics: Written permission from the authors required. The full dataset from the mortality survey will be made available as part of a future publication reporting on the study results. The specific data referenced in this paper are available from the corresponding author on reasonable request. The source code for the RDS Solution can be downloaded from BitBucket (https://bitbucket.org/lshtm-public/rds-api/src/master/, https://bitbucket.org/lshtm-public/rds-ui/src/master/).

## Abbreviations

API: Application Programming Interface
HTML: HyperText Markup Language
LDAP: Lightweight Directory Access Protocol
ODK: ODK [formerly Open Data Kit
PHP: PHP [formerly Hypertext Preprocessor
RDS: Respondent-driven sampling
RTL: Right-to-left
SMS: Short Message Service
SSL: Secure Sockets layer
TLS: Transport Layer Security
UID: Unique Identification
UK: United Kingdom
US: United States
webRDS: Web-based Respondent-driven Sampling
XML: Extensible Markup Language
