# Supplementary material for: Design and implementation of a web-based, respondent-driven sampling solution
Source: BMC Med Inform Decis Mak. 2023 Jul 5;23:113. doi: 10.1186/s12911-023-02217-0 (PMC10320937; doi:10.1186/s12911-023-02217-0)
Supplement: Supplementary file 1 — Supplementary Material 1 [file 12911_2023_2217_MOESM1_ESM.docx]

# SUPPLEMENTAL MATERIAL: List of third-party packages

| **Package** | **Version** | **Function** |
| --- | --- | --- |
| ashallendesign/short-url | 5.1 | Create short urls |
| directorytree/ldaprecord-laravel | 2.4 | LDAP connectivity and authentication |
| firebase/php-jwt | 5.4 | Generation of secure recoverable tokens for survey authentication |
| fruitcake/laravel-cors | 2.0 | Enabling cross origin requests on the API |
| guzzlehttp/guzzle | 7.0 | HTTP layer that sits on top of Curl enabling internal communications with API |
| inertiajs/inertia-laravel | 0.43 | Front end templating |
| laravel-notification-channels/Twilio | 3.1 | Communication with Twilio |
| laravel/framework | 8.5 | PHP Framework in which RDS is built |
| laravel/sanctum | 2.11 | General application authentication interface |
| sentry/sentry-laravel | 2.11 | Application monitoring (error detection) |
| spatie/laravel-permission | 5.5 | Role-based access control |
| vinkla/hashids | 9.1 | Unique IDs for various reasons including creating temporary identifiers |
| Vue | 2/3 | Front end templating framework |
